# Monthly Summary

**Published:** 1898-03

**Authors:** 


### Monthly Summary.

---

THE CARE OF VULCANIZERS.—In spite of the fact that the vulcanizers sold by the reliable manufacturers are submitted to severe tests by hydrostatic pressure—some of them to a pressure of 900 pounds to the inch—explosions have not been unfrequent, generally due to the ignorance of the assistants who watch them, or pretend to watch them, during the process of vulcanization, and not a little to the carelessness of the dentists themselves, who fail to impress upon the students that these little steam boilers require as much attention as boilers of greater capacity. In the first place it does not pay to use cheap vulcanizers any more than cheap German tools and instruments, or the cheaper grades of artificial teeth. In the next place, one should familiarize himself with every part of the machine; realize the importance of keeping it and its belongings clean, and adhere strictly to the rules laid down by the manufacturers. Where thermometers are used, it ought not to be forgotten that too rapid and too great heat at starting, especially if the flame is allowed to surround and reach the top cover, may deceive, and that the thermometer at 320° may really indicate only the temperature of the cover, and not that of the flask inside. The safety disk used on some modern vulcanizers should not be forced on too tight, as this weakens them. The boiler should on no account ever be perfectly full of water; at least one inch or more of steam room should be left above the water. When the heat is first applied, the valve for the escape of steam should be opened for a few minutes to allow a free escape of steam, as this leaves in the boiler, after the valve is closed, an atmosphere of pure steam, and precaution should be taken not to use any more force in closing the valve than is necessary to make it steam-tight. We are warned, too, not to daub too much black lead or soapstone powder about the packing. In fact, it is better never to use any if it can be avoided. Oil should never be used, as it rots the rubber packing, and may become gummy and cement the core to the pot, to the damage of the rubber packing.

Too frequent use of the black lead and soapstone wears away the screw thread. In heating up, the flame should not be larger than will cover the bottom of the boiler. If students can have these simple instructions put into their brains, it may save some of them from having their heads blown off. If they will not attend to them, they deserve to be killed. Thermometers, in the cities, ought to be relegated to the list of things which are obsolete in the laboratory. Proper gas regulators and steam gauges should be used, and dentists owe it to themselves and their students not to neglect every proper precaution in the use of these laboratory demons.—*Indiana Dental Journal.*

---

MAKING PARTIAL DENTURES.—Impressions, either for full or partial duntures, are better obtained with plaster of Paris. Where difficulty of removal is foreseen, I insert a rim of compound which takes the place of the plaster around the teeth. Having run my model and articulated it (when necessary), I very slightly and smoothly trim the plaster around necks of the teeth to insure a close fit. A snug fitting partial plate will not cause the teeth to decay as one does which works closely against them.

With the cast in proper shape we mold a plate on it and mount with teeth of the proper shade, size and shape, and properly set for correct articulation and expression.

I next take some dissolved black rubber of creamy consistency, and smear it over the cast, being careful to rub it well into every part over which I wish the plate to fit. Then gently heat a sheet of rubber and press it evenly into place, and with a hot, sharp knife, trim around the teeth, working the rubber well in between them. The portions of rubber that are to receive the teeth, must, of course, be made thicker; then heat the teeth over the spirit lamp, and press into place.

After the teeth are in proper position, trim with a hot knife all the excess of rubber, putting the plate in as near the desired shape as possible, then flash and vulcanize.

In ordinary cases, from the time your cast is ready till your plate is prepared for the flask does not consume over twenty minutes. Could that be done by the old process of waxing?

Thus you have a saving of time and no waste of rubber, for you are always sure of getting in the proper amount of rubber, and run no risk of getting in too much or too little. The plate is just the desired thickness, and no excess to be scraped away, in fact, I have no scraping to do at all, merely sandpapering the plate and polishing it. Besides this the vulcanite is tougher and better than when thick plates are made. It fits perfectly around the natural teeth without any filing. How many of us have found that in filing a partial plate, to make it fit around the natural teeth, we met a difficulty to know just how far to go that the fit might be perfect? By this method such difficulty is avoided.

By this plan there is no danger of breaking the cast or marring the work in any way.

My reason for using dissolved black rubber is perhaps only a whim, but I like to line my rubber plates with black.

Please do not raise the objection that without the old-style pressure the rubber will not be forced into every part, my experience has taught me differently.

I have tried this method for two years, and will you allow me to state a fact? I have never had one failure since adopting it.—*Dr. D. A. Anderson, in Dental Brief.*

---

THE NUISANCE of tobacco slobber and smoke is expected in the nasty quarters of the abandoned refuge of humanity in our worst cities, but it ought to be a surprise, and is a shame and a disgrace, to see it in our dental colleges! How can we expect gentlemanly deportment, refined manners, chaste language, or even clear intellect, where there is such filth? And how can we look for a clean profession to come from such low morals and lax discipline in its students? —*Dental Brief.*

---

FOR BURNS.—Of all the various remedies recommended for burns, nothing has succeeded so well as bicarbonate of soda, applied wet. It relieves the pain almost instantly and in most cases prevents a blister.

SETTING BRIDGES.—I apply the dam where practicable and coat the pier teeth or roots with nitrate of silver. This may be done by direct application or by cataphoresis. There are three points of advantage in this method:

1st. If the tooth is a live tooth, the sensitive condition is obviated, especially if the cement is slightly acid in reaction.

2d. An oxyphosphate cement will adhere to this surface more surely than to the tooth.

3d. In case any cement washes away at any time, or in case a band does not cover the root entirely, the nitrate of silver coating protects it thoroughly.

I might add a fourth reason, and that is that the tooth would be in an aseptic condition and would have a tendency to render the cement so, but that is conjecture, as I have never removed a bridge that has been set under these conditions.—A. Jameson, *Review.*

---

BE CLEANLY.—Every few days one can hear remarks from patients about some dentist using soiled towels, napkins or rubber dam.

I cannot understand why anyone wants to do it. If they can not make dentistry profitable enough so they can use clean towels, napkins and rubber dam, for the love of their brothers, if not for the profession, get out of it.

Success in dentistry depends largely upon one's ability, combined with cleanliness—i. e., spotless napkins on both tray and headrest, with towels and operating coats from same drawer—one's success is assured.—E. H. L., in *Dental Weekly.*

---

DOCTORS OF MEDICINE who undertake the care of the sick are subject to punishment for malpractice, or for professing qualifications which they do not possess. There is an organized system, however incompletely it succeeds, for the protection of the public against medical incompetents and imposters. But there seems to be no recognized method of ascertaining the competence of a Chrlstian science healer, nor anything to hinder any crank from hanging out a Christian science sign and assuming responsibility for the gravest diseases.—*Life.*

